# Associations of baseline characteristics, patient-reported outcomes, and satisfaction with pain therapy with the patient's global impression of change: a prospective cohort study

**DOI:** 10.1016/j.bja.2026.01.010

**Published:** 2026-02-13

**Authors:** Lydia Roper, Joletta Belton, Claudia Weinmann, Dominique Fletcher, Patricia Lavand'homme, Eija Kalso, Winfried Meissner, Daniela Constanze Rosenberger, Daniel Segelcke, Jan Vollert, Esther Miriam Pogatzki-Zahn

**Affiliations:** 1 Department of Clinical and Biomedical Sciences, Faculty of Health and Life Sciences, University of Exeter, Exeter, UK; 2 NIHR Exeter Biomedical Research Centre, University of Exeter, Exeter, UK; 3 Pain Advocate, Fraser, CO, USA; 4 Jena University Hospital, Department of Anesthesiology and Intensive Care Medicine, Friedrich Schiller University Jena, Germany; 5 Anaesthesia and Intensive Care Department, Ambroise Paré Hospital, APHP, Université Paris-Saclay, UVSQ, Boulogne, France; 6 Department of Anesthesiology and Acute Postoperative & Transitional Pain Service, Cliniques Universitaires Saint Luc - University Catholic of Louvain, Brussels, Belgium; 7 Department of Anaesthesiology, Intensive Care and Pain Medicine, Helsinki University Hospital and University of Helsinki, Helsinki, Finland; 8 Department of Anaesthesiology, Intensive Care and Pain Medicine, University Hospital Muenster, Muenster, Germany

**Keywords:** acute pain, core outcome set, pain assessment, patient-reported outcome measures, psychometric properties, sensitivity-to-change, surgery

## Abstract

**Background:**

Patient-reported outcome measures (PROMs) are key elements of assessing the efficacy of perioperative pain management. Here, we aimed to capture the association of 10 individually reported aspects of patient's specific impression of change since surgery (PSIC) related to four outcome domains of a previously defined core outcome set, relative to the patient's global impression of change (PGIC). We further evaluated the influence of type of surgery, sex, preoperative baseline characteristics, and satisfaction with pain management on PGIC.

**Methods:**

This exploratory analysis used the PROMPT NIT-1 study data (2661 patients, 18 sites, four surgery types: total knee arthroplasty, sternotomy, breast cancer surgery, or endometriosis surgery). Male and female adults were included. All PROMs were assessed on postoperative day 3. We used ordinal regression models with PGIC as a dependent variable and PSICs as independent variables.

**Results:**

The overall model achieved a pseudo-R^2^ of 0.55 (relative domain contributions: pain intensity 55%, self-efficacy 19%, adverse events 15%, and pain-related interference of physical functioning 10%). Pain-related worrying and depression had no association with the PGIC, whereas anxiety, preoperative pain, opioid intake, low satisfaction with and wish for more treatment, low treatment agency, and overall dissatisfaction were associated with less improvement after surgery. Receiving information about treatment was associated with greater improvement on the PGIC.

**Conclusions:**

Although all four domains contributed to PGIC after surgery, pain intensity was the most important. These findings highlight the importance of both managing postoperative pain and optimising patient experience by addressing self-efficacy, adverse events, and pain-related interference of physical functioning.

**Clinical trial registration:**

NCT 03834922

**Editor’s key points**

• Patient-reported outcomes, such as the effect of pain on physical and emotional status, are not routinely measured in clinical trials or clinical practice. This might contribute to inadequate pain management from the patient’s perspective.
• In this subanalysis of data from a large prospective cohort study, the authors aimed to determine how specific impressions of change contribute to the patient's global impression of change in pain after total knee arthroplasty, sternotomy, breast cancer surgery, and endometriosis surgery.
• Pain intensity was the most important outcome from patients’ perspective, but the other three domains (physical function, adverse effects, and self-efficacy) remained relevant during all surgeries and in both male and female patients.
• Assessing patient-reported outcomes is vital in clinical trials and also clinical practice.

Effective postoperative pain management is critical for functional recovery and alleviating patient suffering. However, pain often is not treated optimally.^1^ Suboptimal management of acute pain is associated with impaired physical function, prolonged recovery, increased risk of persistent opioid use, and the development of chronic postsurgical pain.^1^

One avenue for improving postoperative pain management is to monitor patient-reported outcome measures (PROMs). PROMs are key elements of postoperative pain management as they assess the subjective experience of pain of the patient. However, there has been a lack of consensus both on what to measure, as evidence suggests that pain intensity alone is inadequate,^2^ and what would be the best method to measure it, as the existence of many PROM tools make comparability between studies unfeasible.

Given the many existing PROMs with often overlapping scope, core outcome sets,^3^^,^^4^ which are recommended to be used in every trial, clinical practice, or both to capture the most relevant aspects related to a certain disease, have recently been developed.^5^ If this minimum set of outcomes were assessed in every trial, these trials would be more meaningful, firstly by addressing the most relevant outcomes and, secondly by being more comparable with each other, enhancing the robustness and generalisability of study results and facilitating systematic reviews and meta-analyses.

The Innovative Medicines Initiative (IMI)-PainCare subproject, PROMPT (Providing Standardised Consented PROMs for Improving Pain Treatment), aimed at improving the management of acute and persistent postoperative pain by identifying a core set of PROMs that are predictive indicators of treatment success, both for clinical practice and randomised controlled trials. To address what should be measured, IMI-PainCare defined a core outcome set of outcome domains to assess postoperative pain (including pain intensity at rest and pain during activity, physical function, adverse events, and self-efficacy) in an international consensus process including researchers, clinicians, and people with lived experience.^3^ Next, to assess how to measure this core outcome set, another consensus process was performed to identify suitable domain-specific PROMs, also assessing their psychometric properties and sensitivity to change.^4^^,^^6^

What remains unclear is how people weigh individual aspects of their experience within the patient's global impression of change (PGIC). Therefore, this study aimed to quantify how the patient's specific impression of change (PSIC) within the PainCare PROMPT core outcome set^3^ contribute to PGIC on postoperative day (POD) 3, and to examine how surgery type, sex, preoperative baseline characteristics, and satisfaction with perioperative pain management are associated with PGIC.

## Methods

This is an exploratory analysis of the PROMPT-NIT 1 study,^6^ where the methods are described in detail. Below is a short summary of the study along with the specific details of this investigation.

### Study design

The PROMPT-NIT study was a multicentre prospective cohort study collecting PROMs and clinical data from patients before and after surgery. The aim was to investigate sensitivity to change of specific PROMs for the assessment of postoperative pain outcomes. The study was performed in accordance with the Declaration of Helsinki. Ethics approval was granted by the ethics committee of Jena University Hospital, Jena, Germany (Ref. 2019-1298-Bef, dated February 6, 2019) and relevant local ethics committees for each site. All patients gave written informed consent. Reporting adheres to the Strengthening the Reporting of Observational Studies in Epidemiology (STROBE) guidelines, and the STROBE checklist can be referred to in Supplementary material. The current analysis was an exploratory one that was prompted by discussions of the primary findings of the PROMPT-NIT study and was therefore not part of the study registration.

### Eligibility criteria

Across 18 sites, adult female and male patients (>18 yr old) undergoing four different elective inpatient surgical procedures (total knee arthroplasty, sternotomy, breast cancer surgery, endometriosis surgery) were eligible to take part in the study. Patients were excluded if they were unable to give consent, for example owing to cognitive impairment, or if questionnaires were not available in the language in which the patient was fluent.

### Procedure

At baseline, after informed consent, data on patient characteristics, comorbidities, and preoperative treatment with opioids and other analgesics were collected. Other relevant parameters, including quality of life, depression and anxiety, pain sensitivity, pain expectancy, pain-related worrying, preoperative pain, and neuropathic qualities of preoperative pain, were also collected. PROMs were completed on POD 3, including patients’ PGIC and PSICs.

### Primary and secondary outcomes

The primary outcome was the impact of each PSIC element on the pseudo-R^2^ of the regression models of PGIC. Secondary outcomes were impact of baseline characteristics on PGIC and association of satisfaction with pain treatment aspects on PGIC. The PGIC was captured by the following question: ‘Since the start of the study, my overall status is’: with a seven-point Likert scale, labelled ‘very much improved’, ‘much improved’, ‘minimally improved’, ‘no change’, ‘minimally worse’, ‘much worse’, and ‘very much worse’.

The 10 PSIC items used the same seven-point scale, with the following lead questions: ‘Since the first day after surgery until now, how would you describe the change (if any) in…” (1) your pain intensity at rest while lying in bed?; (2) your pain intensity while lifting your extended arm sideways on the operated side/changing from the lying position to sitting upright/taking a deep breath/bending your operated knee?∗; (3) your worst pain intensity while doing physiotherapy?; (4) your average pain intensity during the last 24 h?; (5) your worst pain intensity during the last 24 h?; (6) the interference of doing activities in bed such as turning, sitting up, changing position because of pain?; (7) the interference with doing physiotherapy owing to pain?; (8) the interference with lifting your extended arm sideways on the operated side/changing from the lying position to sitting upright/deep breathing/bending your operated knee owing to pain?∗; (9) your belief of being able to be active and do your tasks, even though you have still been handicapped by pain after your surgery?; and (10) your symptoms such as fatigue, dizziness, nausea, or similar symptoms?

The items marked with ∗ were specific for each surgery and are given in the following order: breast cancer surgery/endometriosis surgery/sternotomy/total knee arthroplasty.

### Statistical analyses

To investigate the contribution of each PSIC on PGIC, we conducted a forward stepwise-inclusion multiple ordinal regression analysis with PGIC as a dependent variable and the PSICs (items 1–10 above) as potential independent variables. We chose the best model based on the Akaike information criterion. Seven models were constructed using data from: (1) all patients; (2) all patients undergoing total knee arthroplasty; (3) all patients undergoing sternotomy; (4) all patients undergoing breast cancer surgery; (5) all patients undergoing endometriosis surgery; (6) all female patients; and (7) all male patients. We used pseudo-R^2^ (Nagelkerke’s method) to assess the model fit.

To investigate the impact of baseline characteristics on PGIC, we constructed ordinal logistic regression models for the following parameters, corrected for patient age decade at the time of surgery, patient sex, and surgery type: (1) depressive symptoms (Hospital Anxiety and Depression Scale [HADS] depression score elevated, yes/no); (2) anxious symptoms (HADS anxiety score elevated, yes/no); (3) elevated pain-related worrying (Pain Catastrophizing Scale elevated, yes/no); (4) patient reporting pain before surgery (yes/no); and (4) patient receiving opioid treatment before surgery (yes/no).

To investigate the relationship between satisfaction with pain treatment and PGIC, we used ordinal logistic regression models for the following parameters, corrected for patient age decade at the time of surgery, patient sex, and surgery type: (1) satisfaction with pain treatment (‘Please mark beside the one number that best shows how satisfied you are with the results of your pain treatment since your surgery’, 0–10 Likert scale with 0=extremely dissatisfied and 10=completely satisfied); (2) treatment agency (‘Were you allowed to participate in decisions about your pain treatment as much as you wanted to?’, 0–10 Likert scale with 0=not at all and 10=very much so); (3) treatment information (‘Did you receive any information about your pain treatment options?’, yes/no); and (4) treatment sufficiency (‘Would you have liked more pain treatment than you received?’, yes/no).

For each of the above regression models, odds ratios (ORs) along with 95% confidence intervals (CIs) were calculated. Despite this being a multicentre study, we did not include centre as a variable in the regression models, as ordinal logistic regression models are less likely to converge with more variables included.

## Results

### Patient characteristics

Patient characteristics, including patient flow through the study, have been described previously.^6^ In this analysis, a total of 2661 participants were included. Of these, 510 (19%) underwent total knee arthroplasty, 972 (37%) underwent sternotomy, 484 (18%) underwent breast surgery (conservation or mastectomy), and 695 (26%) underwent endometriosis surgery (laparoscopy, complex surgery, hysterectomy). The mean (range) age of all patients was 54 (18–91) yr. The sample was predominantly female (1718, 65%) as it was biased by breast cancer and endometriosis surgery where only female patients were included.

### Descriptive patients’ global impression of change

**Fig 1:**
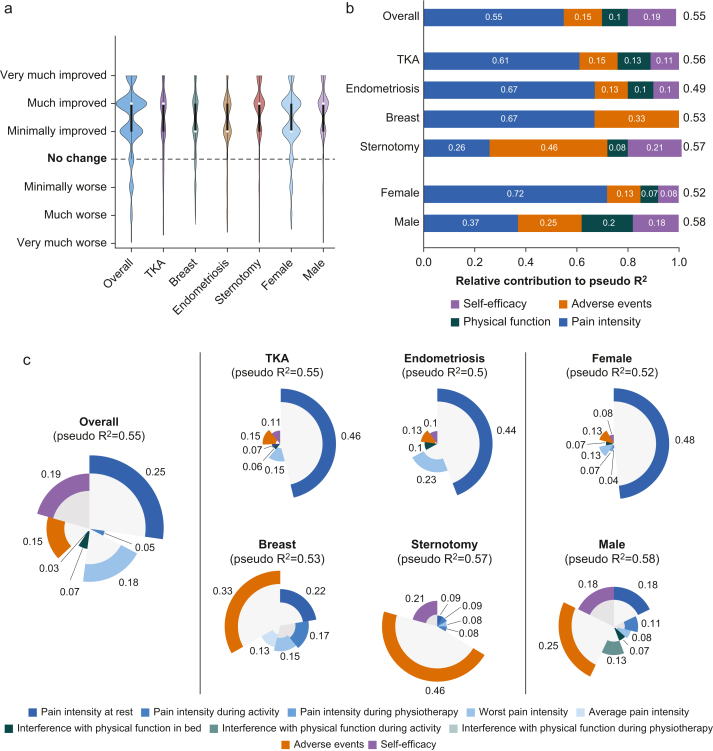
Patients’ global impression of change (PGIC) and contributing patient-reported outcome measures (PROM) domains. (a) PGIC on day 3 by overall, by surgery type (total knee arthroplasty [TKA], breast surgery, endometriosis-related surgery, sternotomy), and by sex; medians were ‘much improved’ except ‘minimally improved’ for breast surgery and endometriosis. Data are displayed in violin plots with 5–95% confidence intervals, interquartile ranges (black box), and medians (white dot). (b) Relative contribution of pain, self-efficacy, interference, and adverse events to the pseudo-R^2^ overall, by surgery, and by sex; pain contributed most except in sternotomy (adverse events) and in males (lower pain contribution). Data are displayed in stacked bar plots. (c) Relative weights of individual change items from all four PROM domains included as predictors in the multiple regression models, shown by overall, by surgery, and by sex. Slice size reflects the proportion of each item’s relative contribution to the model’s total pseudo-R^2^. Data are displayed in pizza plots representing relative contributions to the models total pseudo-R^2^.

The overall PGIC for the full sample and per surgery is displayed in Figure 1a. On the seven-item scale, the overall median response on POD 3 was ‘much improved’ (interquartile range one-item wide). Per surgery, the median response was ‘much improved’ for total knee arthroplasty and sternotomy, and ‘minimally improved’ for breast cancer and endometriosis surgery.

### Contribution of patient-reported outcome measure domains and subdomains to patients’ global impression of change

The optimal model included reported changes in pain intensity, interference with physical function, adverse events, and self-efficacy, and found an overall pseudo-R^2^ of 0.55. Within this model, change items for pain intensity contributed 55% of the total pseudo-R^2^, self-efficacy 19%, change in adverse events 15%, and interference with physical function owing to pain-related change items 10% (Fig. 1b, top row). For each type of surgery, the contribution of each domain to the PGIC varied, with pain intensity usually being the highest contributing domain, with 67% for breast cancer and endometriosis surgery and 61% for total knee arthroplasty. Only for sternotomy, the leading contributor was adverse effects with 46% (Fig. 1b). Similarly, when splitting by sex, pain intensity contributed 72% of pseudo-R^2^ in females but only 37% of pseudo-R^2^ in males (Fig. 1b). In all models except one, PSICs from all four domains were included as significant predictors; this exception was breast cancer surgery, which only included as predictors adverse events (33%), pain at rest (22%), pain during movement (17%), worst pain (15%), and average pain (13%, Fig. 1c).

### Association of baseline characteristics and patients’ global impression of change

Of the parameters used to correct all models, surgery, but not age decade or sex, exhibited an influence on the PGIC. Patients undergoing sternotomy on average reported higher improvement than other surgeries. Preoperative elevated scores for pain-related worrying or depression had no effect on PGIC, whereas preoperative anxiety, pain, or opioid intake all led to less improvement on the PGIC scale (see Table 1 for all ORs).

**Table 1:** Association between baseline features and patients’ global impression of change. CI, confidence interval; HADS, Hospital Anxiety and Depression Scale; OR, odds ratio; PCS, Pain Catastrophizing Scale.

Parameter	OR (95% CI)	Direction of effect
Elevated PCS score	1.17 (0.92–1.50)	No effect
Elevated HADS depression score	1.31 (0.96–1.80)	No effect
Elevated HADS anxiety score	1.27 (1.02–1.58)	Anxiety: less improvement
Preoperative pain	1.27 (1.04–1.54)	Pain: less improvement
Preoperative opioid intake	1.47 (1.06–2.04)	Opioids: less improvement

### Relationship of patients’ global impression of change and satisfaction with pain treatment

Patients’ satisfaction with pain treatment was clearly reflected in PGIC. Patients who reported having wanted to receive more pain treatment reported less improvement in PGIC. Conversely, patients who reported receiving information about pain treatment options reported more improvement in PGIC. Similarly, reporting low levels of agency over pain treatment was associated with lower levels of reported improvement in PGIC. Finally, overall dissatisfaction with pain treatment was associated with lower levels of improvement in PGIC (see Table 2 for all ORs).

**Table 2:** Association of patient-reported satisfaction with and agency over treatment with patients’ global impression of change. CI, confidence interval; OR, odds ratio.

Parameter	Score	OR (95% CI)	Direction of effect
Wish for more treatment		2.40 (1.95–2.94)	More treatment: less improvement
Information of treatment		0.64 (0.54–0.74)	Information given: more improvement
Agency over treatment	0	2.86 (2.24–3.66)	No agency: less improvement
	1	2.72 (1.59–4.65)	
	2	1.92 (1.18–3.14)	
	3	1.93 (1.23–3.02)	
	4	2.28 (1.39–3.72)	
	5	2.16 (1.59–2.94)	
	6	2.51 (1.65–3.82)	
	7	1.47 (1.08–2.01)	
	8	1.29 (1.00–1.68)	
	9	1.09 (0.86–1.39)	
	10	1	
Satisfaction with treatment	0	3.12 (1.23–7.96)	Dissatisfaction: less improvement
	1	2.68 (0.92–7.78)	
	2	5.41 (2.79–10.50)	
	3	5.70 (3.31–9.81)	
	4	9.21 (5.47–15.51)	
	5	4.83 (3.45–6.78)	
	6	3.59 (2.47–5.22)	
	7	3.18 (2.41–4.18)	
	8	2.39 (1.88–3.03)	
	9	1.79 (1.46–2.19)	
	10	1	

## Discussion

### Composition of patients’ global impression of change

First, we investigated how the four domains studied here (pain intensity, interference with physical function, adverse events, and self-efficacy) contribute to PGIC. Although all four domains contributed significantly to PGIC, measures of pain intensity—especially pain at rest—had by-far the largest effect, contributing more than half of the total pseudo-R^2^ of the global impression. This could indicate that pain intensity measures, especially pain at rest, reflect the patients' most relevant state of change in the days after surgery. However, domains such as self-efficacy, adverse events, and, to a lesser degree, interference with physical function, also seem to be relevant to patients. This is intriguing because it indicates that pain intensity, the most frequently used outcome domain in acute perioperative pain trials, is an important outcome from the patients’ perspective, but all four domains remain relevant for the full picture and need to be better addressed to effectively treat pain after surgery. Despite this, in clinical trials, pain intensity is assessed regularly for pain management after surgery, as shown, for example for total knee arthroplasty^7^ or sternotomy.^8^ This is one of the reasons why current studies do not allow sufficient judgements related to efficacy and acceptance of the investigated treatment approach.

### Impact of the type of surgery for patients’ global impression of change

We found variation between surgeries in contributing factors. Although some of this variation should be ascribed to random scatter, a marked difference was that a change in adverse events appeared to be most relevant for patients after sternotomy and breast cancer surgery, whereas a change in pain intensity was most relevant for total knee arthroplasty and endometriosis surgery. There may be several reasons for this. First, pain intensity was higher in patients after total knee arthroplasty and endometriosis surgery, and a reduction of pain intensity might be more relevant for these surgeries.^6^ Second, different surgeries are treated differently^9^ and the type of treatment is responsible for the adverse events and side-effects. Milder adverse events might mean a reduction in these is seen as less relevant to patients’ PGIC. These differences demonstrate from the patient perspective why measuring multiple outcome domains is important for capturing the full experience of patients across surgeries.

### Patient sex and patients’ global impression of change

Another interesting aspect was that PGIC did not differ between sexes, but the composition within PGIC did (Fig. 1). For females, pain intensity was most relevant, whereas for male patients, it was more a composition of several factors with adverse events being a little more prominent than other domains. One reason for this might be the higher pain intensity females have compared with males after surgery. This has been shown earlier in many studies^10^^,^^11^ leading to a more prominent role of pain intensity, and a change contributing to pain intensity might therefore be more relevant. However, females are more prone to side-effects and adverse events from drugs, such as opioids, with more frequent postoperative nausea and vomiting.^12^^,^^13^ Thus, it is more likely that the role of adverse events for an improvement might be taken more seriously by males than by females. This has not been investigated in detail and needs further assessments. Together, however, all four outcome domains are relevant for both males and females, implying all domains should be collected in interventional trials and clinical practice.

### Impact of baseline characteristics on patients’ global impression of change

We also investigated the impact of several baseline clinical and psychological characteristics on PGIC. Overall, although elevated scores for pain-related worrying and depression had no effect on PGIC score, elevated levels of anxiety, pain, and opioid intake all led to less improvement in PGIC. The lack of effect of pain-related worrying and depression in our results is notable, as previous literature has shown associations between these factors and various negative surgical outcomes, such as increased postoperative pain severity,^14^, ^15^, ^16^ the incidence of chronic pain,^17^ more analgesia requests and chronic opioid use,^15^^,^^18^ and poorer quality of life.^17^ Conversely, our results suggest that anxiety is relevant for the perception of overall improvement after surgery. Similar results have indicated that anxiety is associated with poorer acute pain control after surgery^14^^,^^19^ and is the main psychological risk factor for the development of chronic postsurgical pain.^20^^,^^21^ Taken together, it seems as though anxiety is particularly important in the perioperative period by affecting patients’ impression of improvement, whereas the effects of depression and pain-related worrying are less consistent. This being said, the direction of effect was similar for all psychological characteristics in our study, so we should not refute depression or pain-related worrying as important, and potentially modifiable, predictors of surgical outcomes. For the clinical characteristics, our findings are consistent with previous studies also reporting that preoperative pain and preoperative opioid intake are associated with poorer pain and surgical outcomes.^14^^,^^22^ It should be noted that ORs for all tested parameters were in a similar range (1.17–1.47), with lower boundaries of CIs of 0.92–1.06, indicating that on the basis of these findings, we should not make strong conclusions about relevance or irrelevance of either of the predictors.

### Relationship of patients’ global impression of change and satisfaction with pain treatment

Finally, our results indicate that patients’ satisfaction with pain treatment was clearly reflected in PGIC. Overall, patients who reported wanting to receive more pain treatment, low agency over their pain treatment, and overall dissatisfaction with their pain treatment reported lower levels of improvement in PGIC. Conversely, patients who reported receiving information about pain treatment options reported greater improvement in PGIC. These findings align with previous evidence from analyses of registry studies, which similarly highlight the importance of providing patients with sufficient information of their treatment options, participation in pain treatment decisions, and perceived adequacy of pain relief and desire for more treatment.^23^^,^^24^ One should note, however, that from a lived experience perspective the factors underscoring satisfaction are quite nuanced. For example, although information about pain treatment options is important, it should be tailored to the individual patient, for example by considering factors such as a family history of opioid dependence. Additionally, although shared decision-making is important, this should not undermine the value of support and reassurance from healthcare professionals. Overall, these results highlight that factors other than pain intensity are relevant to patient satisfaction, so by better addressing these we can improve not only overall satisfaction but also the perception of global improvement.

### Limitations of the study

There are some limitations inherent in our study. As an exploratory approach, these findings should be seen as indicative rather than confirmative. There are overlaps between subgroups: all patients undergoing endometriosis-related or breast-cancer surgery were female, so we cannot clearly distinguish between the effects of sex and surgery. All humans are subject to recall bias: the changes here are perceived on POD 3, not measured as contrasts from the day after surgery. Lastly, the inclusion of 18 centres across 10 countries means that the associations observed here could also be influenced by differences in perioperative pain management between centres.

### Conclusions

Our analysis shows that all four domains of a previously proposed core outcome set^3^^,^^4^ (pain intensity, interference with physical function, adverse events, and self-efficacy) are relevant for patients to determine improvements in perioperative pain. Although all domains are relevant, pain intensity seems to be the most important domain when considering global improvement. Nevertheless, PROMs for each domain should be included in trials for acute perioperative pain management to get the full picture. Results of PROMs should be better addressed in a concerted way to treat pain after surgery in individual patients in the future.

## Funding

The Innovative Medicines Initiative 2 Joint Undertaking [grant agreement no 777500 to EMPZ]. This Joint Undertaking receives support from the European Union's Horizon 2020 research and innovation programme and EFPIA www.imi.europa.eu; www.imi-paincare.eu; The National Institute for Health and Care Research Exeter Biomedical Research Centre and National Institute for Health and Care Research Exeter Clinical Research Facility.

## Authors’ contributions

Study design and management: WM, CW, EPZ

Experiments: DF, PL, EK, WM, DR, EPZ

Data acquisition, handling, and analysis: LR, DS, JV

Figures: LR, DS, JV, EPZ

Manuscript drafting: LR, JV, EPZ

Manuscript revision: all authors

## Declarations of interest

WM has received payments for advisory boards and talks from Grünenthal, Tafalgie, Merck, Sanofi, Kyowa. His institution received research support from European Commission, Federal Joint Committee (GBA), Else-Kröner Fresenius Foundation, Medtronic, Vertanical. JV has received financial support from Viatris for research activities, and consultancy fees from Merz Therapeutics, Grünenthal, and AstraZeneca. EMPZ received financial support from Grünenthal, Germany, for research activities and advisory and lecture fees from Grünenthal, Germany, MSD/MERCK, Germany, and Medtronic, Switzerland. In addition, she receives scientific support from the German Research Foundation (DFG), the Federal Ministry of Education and Research (BMBF), the Federal Joint Committee (G-BA), and the Innovative Medicines Initiative 2 Joint Undertaking under grant agreement No 777500. This Joint Undertaking receives support from the European Union's Horizon 2020 research and innovation program and EFPIA. All money went to the institutions (WWU/UKM) EMPZ is working for. All other authors declare that they have no conflicts of interest.
