# Supplementary material for: Associations of baseline characteristics, patient-reported outcomes, and satisfaction with pain therapy with the patient's global impression of change: a prospective cohort study
Source: Br J Anaesth. 2026 Feb 13;136(4):1341–8. doi: 10.1016/j.bja.2026.01.010 (PMC13014496; doi:10.1016/j.bja.2026.01.010)
Supplement: Multimedia component 1 [file mmc1.docx]

STROBE Statement—checklist of items that should be included in reports of observational studies

|  | Item No. | Recommendation | Page  No. | Relevant text from manuscript |
| --- | --- | --- | --- | --- |
| **Title and abstract** | 1 | (*a*) Indicate the study’s design with a commonly used term in the title or the abstract | 4 | Study title: “Association of baseline characteristics, specific patient-reported outcomes, and satisfaction with pain therapy with the global impression of change; a prospective cohort study” |
|  |  | (*b*) Provide in the abstract an informative and balanced summary of what was done and what was found | 4 | In the abstract methods: “This exploratory analysis used PROMPT-NIT1 study data (2,661 patients, 18 sites, ten countries, four surgery types: total knee arthroplasty, sternotomy, breast-cancer or endometriosis-related surgery). Adults (≥18 years), both male and female patients were included. PROMs including PGIC and PSIC were assessed on postoperative day 3 (POD 3).We used ordinal regression models, and a forward stepwise approach with PGIC as a dependent variable and PSICs as independent variables.”  The abstract results: “Using 10 PSICs related to four domains with PGIC as independent variable, the overall model achieved a Pseudo-R² = 0.55 (relative domain contributions: pain intensity 55%, self-effectiveness 19%, adverse events 15%, and pain-related interference of physical functioning 10%). Although domain weights varied by surgery, each were consistently associated with the PGIC. Pain-related worrying and depression had no association with the PGIC, whereas anxiety, pre-surgical pain and opioid intake, low patient satisfaction with and wish for more treatment, low treatment agency, and overall dissatisfaction were associated with less improvement after surgery, whereas receiving information about treatment was associated with greater improvement on the PGIC.” |
| Introduction | | | |  |
| Background/rationale | 2 | Explain the scientific background and rationale for the investigation being reported | 5 | Rationale and existing literature stated in the introduction section: “What still remains unclear is how people weigh individual aspects of their experience within the global impression” |
| Objectives | 3 | State specific objectives, including any prespecified hypotheses | 5 | Statement at the end of the introduction: “Therefore, this study aimed to quantify how specific PSICs within the PainCare PROMPT Core Outcome Set contribute to PGIC on postoperative day 3, and to examine how surgery type, sex, pre-surgery baseline characteristics, and satisfaction with perioperative pain management are associated with PGIC” |
| Methods | | | |  |
| Study design | 4 | Present key elements of study design early in the paper | 7 | Study design is stated in the first subsection of Methods: “This is an exploratory analysis of the PROMPT- NIT 1 study”  “The PROMPT- NIT study was a prospective, multi-centre observational cohort study" |
| Setting | 5 | Describe the setting, locations, and relevant dates, including periods of recruitment, exposure, follow-up, and data collection | 7 | 18 sites across 10 countries |
| Participants | 6 | (*a*) *Cohort study*—Give the eligibility criteria, and the sources and methods of selection of participants. Describe methods of follow-up  *Case-control study*—Give the eligibility criteria, and the sources and methods of case ascertainment and control selection. Give the rationale for the choice of cases and controls  *Cross-sectional study*—Give the eligibility criteria, and the sources and methods of selection of participants | 7 | The Eligibility Criteria subsection gives details on the eligibility criteria, with surgery-specifics: “Across 18 sites, adult patients (> 18 years old) of both sexes (female and male) undergoing four different elective inpatient surgical procedures (total knee arthroplasty, breast surgery, sternotomy, and surgery related to endometriosis) were eligible to take part in the study. Patients were excluded if they were unable to give consent, for example due to cognitive impairment, or if questionnaires were not available in the language in which the patient was fluent.” |
|  |  | (*b*) *Cohort study*—For matched studies, give matching criteria and number of exposed and unexposed  *Case-control study*—For matched studies, give matching criteria and the number of controls per case |  |  |
| Variables | 7 | Clearly define all outcomes, exposures, predictors, potential confounders, and effect modifiers. Give diagnostic criteria, if applicable | 7-8 | Outcomes defined in the Procedures subsection: “At baseline, after informed consent, data on demographics, comorbidities, and preoperative treatment with opioids and other analgesics were collected. Other relevant parameters, including quality of life, depression and anxiety, pain sensitivity, pain expectancy, pain- related worrying, pre-operative pain, and neuropathic qualities of pre-operative pain, were also collected. PROMs were completed on postoperative day 3 (POD 3), including the patients’ global impression of change (PGIC) item, as well as patients’ specific impressions of change (PSIC).”  In the Primary and Secondary outcomes subsection: “The primary outcome was the impact of each specific impression of change element on the total explained variance of the global impression of change. Secondary outcomes were impact of baseline characteristics on the PGIC and association of satisfaction with pain treatment aspects on the PGIC.” |
| Data sources/ measurement | 8* | For each variable of interest, give sources of data and details of methods of assessment (measurement). Describe comparability of assessment methods if there is more than one group | 7-9 | Data sources were PROM questionnaires administered at baseline and postoperative day 3. Details given in the Procedure subsection.  “PROMs were completed on postoperative day 3 (POD 3), including the patients’ global impression of change (PGIC) item, as well as patients’ specific impressions of change (PSIC)” |
| Bias | 9 | Describe any efforts to address potential sources of bias |  |  |
| Study size | 10 | Explain how the study size was arrived at |  | This is an exploratory analysis of an existing sample; therefore, the sample size was predetermined. The original study was powered on the primary hypothesis of the trial for an effect size of 0.25, 80% power, and an alpha-level of 0.0125 using G*Power, showing a sample size of 265 patients per patient group. The included sample size was 2661. |

Continued on next page

| Quantitative variables | 11 | Explain how quantitative variables were handled in the analyses. If applicable, describe which groupings were chosen and why |  |  |
| --- | --- | --- | --- | --- |
| Statistical methods | 12 | (*a*) Describe all statistical methods, including those used to control for confounding | 8-9 | Details of the statistical analysis were given in the Statistical analyses subsection |
|  |  | (*b*) Describe any methods used to examine subgroups and interactions |  |  |
|  |  | (*c*) Explain how missing data were addressed |  | Participants with missing data were excluded from the analysis |
|  |  | (*d*) *Cohort study*—If applicable, explain how loss to follow-up was addressed  *Case-control study*—If applicable, explain how matching of cases and controls was addressed  *Cross-sectional study*—If applicable, describe analytical methods taking account of sampling strategy |  | Not applicable |
|  |  | (*e*) Describe any sensitivity analyses |  |  |
| Results | | | | |
| Participants | 13* | (a) Report numbers of individuals at each stage of study—eg numbers potentially eligible, examined for eligibility, confirmed eligible, included in the study, completing follow-up, and analysed | 10 | Number of participants were described in the Patient characteristics subsection: “Patient characteristics including patient flow through the study have been described previously (6). In short, in this analysis, a total of n=2,661 participants were included. Of these, n=510 (19%) underwent total knee arthroplasty, n=972 (37%) underwent sternotomy, n=484 (18%) underwent breast surgery (conservation or mastectomy), and n=695 (26%) underwent surgery related to endometriosis (laparoscopy, complex surgery, hysterectomy).” |
|  |  | (b) Give reasons for non-participation at each stage |  | Not applicable |
|  |  | (c) Consider use of a flow diagram |  | Not applicable |
| Descriptive data | 14* | (a) Give characteristics of study participants (eg demographic, clinical, social) and information on exposures and potential confounders | 10 | Described in the Patient characteristics subsection: “The mean age of all patients was 54 years (range: 18–91). The sample was predominantly female (n=1718, 65%) as it was biased by breast cancer and endometriosis-related surgery where virtually only female patients were included. “ |
|  |  | (b) Indicate number of participants with missing data for each variable of interest |  |  |
|  |  | (c) *Cohort study*—Summarise follow-up time (eg, average and total amount) | 7 | Only postoperative day 3 (POD3) in this analysis |
| Outcome data | 15* | *Cohort study*—Report numbers of outcome events or summary measures over time |  |  |
|  |  | *Case-control study—*Report numbers in each exposure category, or summary measures of exposure |  | Not applicable |
|  |  | *Cross-sectional study—*Report numbers of outcome events or summary measures |  | Not applicable |
| Main results | 16 | (*a*) Give unadjusted estimates and, if applicable, confounder-adjusted estimates and their precision (eg, 95% confidence interval). Make clear which confounders were adjusted for and why they were included |  |  |
|  |  | (*b*) Report category boundaries when continuous variables were categorized |  |  |
|  |  | (*c*) If relevant, consider translating estimates of relative risk into absolute risk for a meaningful time period |  | Not applicable |

Continued on next page

| Other analyses | 17 | Report other analyses done—eg analyses of subgroups and interactions, and sensitivity analyses |  |  |
| --- | --- | --- | --- | --- |
| Discussion | | | | |
| Key results | 18 | Summarise key results with reference to study objectives |  |  |
| Limitations | 19 | Discuss limitations of the study, taking into account sources of potential bias or imprecision. Discuss both direction and magnitude of any potential bias | 15 | Study limitations were outlined in the limitations subsection: “As an exploratory approach, these findings should be seen as indicative, rather than confirmative. There are overlaps between subgroups: all patients undergoing endometriosis-related or breast-cancer surgery were of female sex, so we cannot clearly distinguish between the effects of sex and surgery. All humans are subject to recall bias: the changes here are perceived on day 3, not measured as contrasts from the day after surgery. Lastly, the inclusion of 18 centres across 10 countries means that the associations observed here could also be influenced by differences in perioperative pain management between centres.” |
| Interpretation | 20 | Give a cautious overall interpretation of results considering objectives, limitations, multiplicity of analyses, results from similar studies, and other relevant evidence | 15 | The conclusions section provides an overall interpretation of results: “Our analysis shows clearly that all 4 domains of a previously proposed Core Outcome Set (3,4) (pain intensity, physical function, adverse events and self-effectiveness) are relevant for patients to determine improvements in perioperative pain. However, whilst all domains are relevant, pain intensity seems to be the most important domain when considering global improvement. Still, PROMs for each domain should be included in trials for acute perioperative pain management to get the full picture.” |
| Generalisability | 21 | Discuss the generalisability (external validity) of the study results |  |  |
| Other information | |  | | |
| Funding | 22 | Give the source of funding and the role of the funders for the present study and, if applicable, for the original study on which the present article is based | 2 | Funding statement provided |

*Give information separately for cases and controls in case-control studies and, if applicable, for exposed and unexposed groups in cohort and cross-sectional studies.

**Note:** An Explanation and Elaboration article discusses each checklist item and gives methodological background and published examples of transparent reporting. The STROBE checklist is best used in conjunction with this article (freely available on the Web sites of PLoS Medicine at http://www.plosmedicine.org/, Annals of Internal Medicine at http://www.annals.org/, and Epidemiology at http://www.epidem.com/). Information on the STROBE Initiative is available at www.strobe-statement.org.
